# IgE-Mediated Legume Allergy: A Pediatric Perspective

**DOI:** 10.3390/jpm14090898

**Published:** 2024-08-25

**Authors:** Carla Mastrorilli, Fernanda Chiera, Stefania Arasi, Arianna Giannetti, Davide Caimmi, Giulio Dinardo, Serena Gracci, Luca Pecoraro, Michele Miraglia Del Giudice, Roberto Bernardini

**Affiliations:** 1 Department of Pediatrics, University Hospital Consortium Corporation Polyclinic of Bari, Pediatric Hospital Giovanni XXIII, 70124 Bari, Italy; 2 Pediatric Unit, Giovanni Paolo II Hospital, ASP Catanzaro, 88046 Lamezia Terme, Italy; 3 Area of Translational Research in Pediatric Specialities, Allergy Unit, Bambino Gesù Children’s Hospital, IRCCS, 00165 Rome, Italy; 4 Pediatric Unit, IRCCS Azienda Ospedaliero-Universitaria di Bologna, 40138 Bologna, Italy; 5 Allergy Unit, CHU de Montpellier, Université de Montpellier, 34295 Montpellier, France; 6 IDESP, UMR A11, Université de Montpellier, 34093 Montpellier, France; 7 Department of Woman, Child and General and Specialized Surgery, University of Campania ‘Luigi Vanvitelli’, 80129 Naples, Italy; giulio.dinardo@studenti.unicampania.it (G.D.); michele.miragliadelgiudice@unicampania.it (M.M.D.G.); 8 Pediatrics and Neonatology Unit, Maternal and Child Department, San Giuseppe Hospital, Azienda USL Toscana Centro, 50053 Empoli, Italy; 9 Pediatric Unit, Department of Surgical Sciences, Dentistry, Gynecology and Pediatrics, University of Verona, 37126 Verona, Italy

**Keywords:** legume allergy, IgE-mediated food allergy, anaphylaxis, cross-reactivity

## Abstract

Legumes are an inexpensive and essential protein source worldwide. The most consumed legumes include peanuts, soybeans, lentils, lupines, peas, common bean and chickpeas. In addition, the food industry is growing interested in expanding the use of legumes to partially replace or substitute cereals. Legumes were described to cause IgE-mediated allergies, and their growing use may also increase the incidence of allergy. The epidemiology of legume allergy varies by region; peanuts and soybeans are the legumes most involved in food allergies in Western countries, whereas lentils, peas, and chickpeas are reported as culprit allergens mainly in the Mediterranean area and India. This review, edited by the Italian Society of Pediatric Allergology and Immunology, summarizes the scientific literature on legume allergy in children and proposes a diagnostic workup and therapeutic approach.

## 1. Introduction

Legumes are an important source of proteins with high biological value and represent a basic food source in many countries. They can be consumed in both fresh and dried forms. The most common legumes cultivated for human consumption comprise lentils, lupines, peanuts, soybeans, broad beans, French beans, peas, chickpeas, cowpeas, and moth beans. Indian pea, fenugreek, alfalfa, Peruvian balsam, tamaridus, Judas tree, Arabic gum, and Copaiba balsam are also legumes. Legumes contain various vitamins, including B vitamins and minerals, including iron, magnesium, potassium, and zinc, as well as phytochemicals, including anthocyanins, phenolic acids, flavonols, and proanthocyanidins [1]. Regarding iron, legumes largely contain non-heme iron, which is less bioavailable than heme iron (commonly found in animal products). Consuming legumes in combination with Vitamin C sources may improve iron bioavailability [2]. At the same time, they also contain anti-nutritional factors (lectins, saponins, protease inhibitors, and phytates) eliminated or reduced in concentration during processing (soaking and cooking). Legumes are also important fodder sources, and their wood, tannins, oils, and resins are employed to manufacture varnishes, dyes, and drugs [3]. Legumes are dicotyledóneas plants of the order of the Fabales. They include six subfamilies, including Cercidoideae, Detarioideae, Duparquetioideae, Dialioideae, Caesalpinioideae, and Papilionoideae. The Papilionoideae subfamily is the largest one and includes the main legumes responsible for allergic reactions (lentils, beans, peas, peanuts, soybeans, and lupines). They are a frequent cause of allergy, especially in childhood, and the fifth most frequent cause of food allergy in Spanish children younger than five years old [3]. The allergens are characterized by resistance to thermal, chemical, and proteolytic denaturalization. Peanuts and soybeans are two of the “big nine” foods that account for the most significant food allergies in the US and Europe and are identified among major food allergens [4,5]. Other legumes, such as lupine, have been increasingly used in Europe due to their nutritional value, health benefits, and sustainable production. According to the recent alert from the Allergy-Vigilance Network, it is considered a potential hidden food allergen responsible for anaphylaxis [6]. Among legumes, there exists an immunological cross-reactivity due to the homology of structure and common antigenic epitopes shared [7]. In most cases, subjects were found to be sensitive or to react to more than one legume. For example, cross-reactivity among lentils, chickpeas, and peas in the Mediterranean area was reported [8]. Furthermore, sensitization to other legumes was also observed in peanut-allergic children, namely fenugreek, lentil, soy, pea, lupine, chickpea, broad bean, and bean [9]. Even though peanut-allergic subjects may present an allergy to other legumes, the percentage of clinically relevant cross-reactivity is much lower than cross-sensitization [10]. Moreover, clinical reports frequently describe an association of peanut allergy with tree nuts, seeds, fruits, and pollen allergies [11]. Currently, the European Food Safety Authority Allergen (EFSA) labeling regulations require the declaration of peanut, soybean, and lupine as allergens in food products. This review presents an update on allergy to legumes in children, including allergen characterization, clinical and diagnostic aspects, natural history, and therapeutic approach.

## 2. Epidemiology

Food allergy affects 3–10% of children, up to 10% of adults, and 40% of children with FA to multiple foods [12,13]. The European Anaphylaxis Registry reported peanuts as the most common food inducing anaphylaxis in children. Soy is commonly involved in allergic reactions. Among other legumes, lupine and pea are responsible for anaphylaxis [14,15]. Regional differences in legume consumption are related to the prevalence of legume allergy, sensitization, and cross-reactivity between legumes. Peanut allergy is the most common legume allergy reported in the UK, France, Switzerland, and North America. Soy allergy is predominant in Japan. Lupine allergy is present in various European countries, while lentil and chickpea allergy is prevalent in Spain [16,17]. Peanut allergy is among the most common allergies, affecting about 25% of children with food allergies [18]. The general prevalence of peanut allergy, based on the OFC, in children in Europe is 0.2–1.6%, and in the United States, it is approximately 2% [19,20]. The reported prevalence of soy allergy is about 0.6% for the adult population, 0.4% for pediatric age, and 2.7% for sensitized children [20,21,22]. Lupine allergy mostly involves subjects with allergies to other legumes, particularly peanuts, but it has also been reported as a primary food allergy. The prevalence of lupine allergy is unknown, but it could be underestimated as lupine may act as a hidden allergen. Lupine sensitization affects 15–20% of subjects with peanut allergy, 6% of atopic subjects, and about 2% of non-atopics, with differences related to geographical area and dietary habits [22,23]. Recently, the Allergy Vigilance Network described lupine as responsible for 2.3% of total anaphylaxis and 1.5% in pediatric age (<18 years) in France over the two last decades, even though in Europe, in the same period, lupine was identified as the elicitor of 0.8% of anaphylaxis [6,24,25]. Previous studies reported a prevalence of lupine allergy in children, with between 6.5% of children having severe food-induced anaphylaxis and 16.7% of children having peanut allergy [26,27]. Peanut, soy, and lupine are considered priority legume allergies in Europe, for which mandatory labeling is required [28]. Non-priority legumes are those not included in the list; tough labeling requirements differ between countries. The prevalence of non-priority legume allergy (chickpea, pea, lentil, and others) is estimated at ≤0.5% in studies exploring the cause of food-induced anaphylaxis and in children with symptoms of food-protein-induced enterocolitis syndrome (FPIES). In contrast, sensitization affects 40% of subjects with known food hypersensitivity to any food allergen [15,29]. Beans, peas, and lentils are among the top 10 allergens responsible for FA, especially among non-Caucasian children and those living in the Mediterranean area [30]. Even in Asian countries, particularly India, legume allergy (lentils and chickpeas) is prevalent in children <15 [31]. In Mediterranean countries, where some legumes (lentils, chickpeas, peas, and beans) are introduced during the first year of age, lentils are the most common cause of legume allergy (80%), followed by chickpeas (59%) and peas (50%). Green beans and white beans represent the legumes better tolerated [32]. The prevalence of probable food allergy to lentils in children is 0.5% (Spain and Greece), while sensitization to lentils concerns 5.3% of a cohort of school-age children from the general population [33]. The prevalence of pea allergy is unknown; sensitization to the pea is 2% in non-atopic subjects and 14% in atopic subjects of a German adult cohort [23,34]. A previous study from Spain, in a population of children with FA, reported a clinically relevant allergy to peas in 2.3%, lentils in 5.9%, and chickpeas in 3.8% of subjects [35]. The pea is also involved in food-dependent exercise-induced anaphylaxis (FDEIA) in a small number of subjects from a study on adults and children with FDEIA [36]. Moreover, many studies showed anaphylaxis to non-priority legumes, specifically lentils and peas, often in conjunction with an allergy to peanuts or other legumes [37,38,39]. A French study on 195 peanut-allergic children demonstrated sensitization to at least another specific legume in 63.9%. Specifically, fenugreek was the most common, followed by lentil, soy, pea, lupine, chickpea, broad bean, and bean. Moreover, FA to at least one legume was demonstrated in 17.4% of the cohort and 27.9% of the sensitized subjects. In the group of sensitized children, 21% were allergic to lentils, 19% to lupine, 15.4% to peas, 9.8% to fenugreek, 8.2% to soy, and 7.4% to chickpeas [9]. The prevalence of chickpea allergy is unknown, but it was reported in 7.7% of Indian children <15 years from a cohort of patients with self-reported food allergy [31]. Another study on 186 children affected by FA showed that 2.3% were allergic to chickpeas, 3.1% to lentils, and 1.5% to peas [40]. Regarding beans, a study showed a prevalence of self-reported symptoms of 1.8% in a general adult cohort; sensitization to white beans is 7.1% in an adult and pediatric cohort with suspected FA [41,42]. Allergic reactions to broad beans are rare, but a few cases have been reported in Spain and Italy [43,44].

## 3. Classification of Legume Food Allergens: Family and Superfamily Proteins

**Figure 1:**
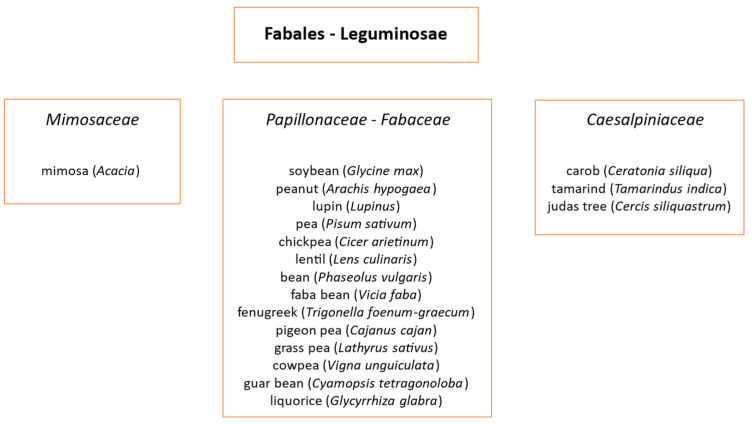
Main taxonomic classes belonging to the order of Leguminosae.

The Leguminosae (Fabaceae) is the third largest angiosperm family after Asteraceae and Orchidaceae (Figure 1).

Edible legumes mainly belong to the Papilionoideae subfamily. This includes soybean, chickpea, bean, pea, licorice, lupine, lentils, and peanut. Some common legumes with potential allergenicity, such as peanut, soybean, lentil, lupine, chickpea, pea, mung, chickpea, red gram, and black gram, have been described by previous reports. Lentils and chickpeas are considered the most allergenic legumes in children living in Mediterranean and Asian countries [7,15,45,46]. Several allergens from different legumes have been identified and characterized. Most belong to the storage proteins family (with two superfamilies, cupins and prolamins), profilins, and pathogenesis-related proteins (PR-10) [7,46]. Most legume food allergens can be gathered into a few families and superfamilies. Cross-reactivity depends on the presence of preserved epitopes accessible to antibodies, and it is described typically between allergens of the same protein family [10]. Numerous allergens belong to the superfamily of prolamines (2S albumins, nonspecific lipid-transfer proteins (nsLTP), α-amylases/trypsin inhibitors, etc.) or the superfamily of cupins (such as 7S and 11S globulins). Still, some legume allergens are homologous to defense proteins or pathogenesis-related (PR) proteins. Then, there are distinct protein families, such as profilins, oleosins, defensins, and other legume allergens [47]. Prolamines encompass seed-storage proteins 2S albumins (Ara h 2, Ara h 6, and Ara h 7 from peanut, Gly m 8 from soy, and Cic a 2S from chickpea) and nsLTP (Ara h 9, Ara h 16, and Ara h 17 from peanut, Pis s 3 from pea, Cic a 3 from chickpea and Len c 3 from lentil). The 2S albumins are highly resistant to heat and pepsin digestion and are associated with severe anaphylactic reactions [47]. The nsLTPs are ubiquitous allergens that are resistant to heat and pepsin digestion and are found in various plant species. Subjects sensitized to nsLTPs may present allergic symptoms to a widespread series of vegetables. The so-called “nsLTP syndrome” ranges from local manifestations, such as oral allergy syndrome (OAS), to anaphylaxis [48]. The cupine superfamily comprises globulin seed-storage proteins that can be classified according to their sedimentation coefficient in 7S globulins (vicilin-type) and 11S globulins (legumin-type). Cupines include allergens from peanuts, Ara h 1 (7S globulin) linked to severe reactions, and Ara h 3 (11S globulin); allergens from soybean Gly m 5 (7S globulin) and Gly m 6 (11S globulin) related to severe reactions; from lentil Len c 1 (7S globulin), from lupine Lup an 1 (7S globulin), from pea Pis s 1 (7S globulin) and Pis s 2 (convicilin), from chickpea Cic a 1 (7S globulin) and Cic a 6 (11S globulin), from fenugreek Tri f 1 (7S globulin) and Tri f 3 (11S globulin), and also from tree nuts (walnut, hazelnut, and cashew) and sesame seeds [47,49]. PR-10 includes a group of proteins with high sequence similarity with the major birch pollen allergen, Bet v 1. PR-10 allergens are present in many species belonging to the Leguminosae (Ara h 8 from peanut and Gly m 4 from soybean), Rosaceae (peach and apricot), and Apiaceae (carrot and celery), families or tree nuts (hazelnut, chestnut). Sensitization to Gly m 4 correlates with severe allergic symptoms to soybeans [50,51]. The profilin family is a pan-allergen labile to heat and pepsin digestion and is identified in pollen, food plants, and latex. Profilin legume allergens include Ara h 5 from peanuts, Gly m 3 from soybean, Lup a 5 from lupines, and Len c 2 from lentils. The profilins and PR-10 allergens are associated with pollen food allergy [10,47]. Additional protein families are oleosins and defensins. Oleosins are resistant to heat and pepsin digestion, recognized in peanuts (Ara h 10, Ara h 11, Ara h 14, and Ara h 15), sesame and hazelnut [52]. They are associated with severe allergic reactions to peanuts [53]. Oleosins have also been described in soybeans, but their allergenic potential is not yet clarified [47]. Further allergens isolated from legumes belong to the defensin family (Ara h 12 and Ara h 13 from peanut and Gly m 2 from soybean). Defensins are involved in severe reactions to peanuts [54].

## 4. Sources of Allergenic Legumes

### 4.1. Peanut

Peanut (*Arachis hypogaea*) is used commercially mainly for oil production but also for peanut butter, snacks, roasted peanuts, extenders in meat product preparations, soups, and cakes. It is high in calories and contains macronutrients (proteins, lipids, and carbohydrates) and micronutrients (vitamins, minerals, and phytonutrients). Peanuts are an important source of protein (about 240 g/kg), essential amino acids, lipids (487 g/kg), and carbohydrates. Peanuts contain 32 different proteins, but only 18 were identified as potential allergens, with Ara h 1, Ara h 2, Ara h 3, and Ara h 6 considered major peanut allergens [21,55]. Moreover, these allergens resist heat, proteolytic enzymes, and chemical denaturation [56]. Many of these proteins are seed-storage proteins and encompass eight different protein superfamilies, namely prolamins, cups, profilin, Bet v 1- like pathogenesis-related (PR)- 10 proteins, lipid transfer proteins (LTPs), defensins, oleosins, and cyclophilins [21]. The seed-storage proteins (2S albumins, 7S globulins, and 11S globulins) represent a clinically relevant marker of sensitization to legumes. According to physical characteristics and solubility, SSP can be divided into two major protein fractions, namely albumins and globulins [56,57]. The two-globulin fractions (7 S and 11 S) include about 87% of the peanut seed proteins. In legumes, these globulin seed-storage proteins are represented by Vicilins (e.g., Ara h 1) and Legumins (e.g., Ara h 3) [57]. Ara h 1 (12–16% of total protein peanut) and Ara h 3 belong to the superfamily. In particular, Ara h 3 has a high sequence homology with glycinin (Gly m 6) from soybeans [58]. The albumin fraction comprises Ara h 2 or Conglutin (2S albumin), which accounts for 6–9% of the overall peanut protein content. Ara h 5 belongs to the profilin family, and Ara h 6 (conglutin family) has 59% homology to Ara h 2. Ara h 7 also belongs to the conglutin family [58,59]. The allergen Ara h 4 is an isoform of Ara h 3, and it was renamed Ara h 3.02 (WHO/IUIS Allergen Nomenclature Sub-Committee, 2017). Meanwhile, Ara h 5 is a minor allergen belonging to the profilin family. Ara h 8 is a pathogenesis-related protein (PR-10) that is homologous to Betv1 and, similarly to profilins, could be involved in pollen food allergy syndrome (PFAS). Nonspecific lipid-transfer (nsLTPs) proteins include Ara h 9, Ara h 16, and Ara h 17, which are pan-allergens and may be responsible for the so-called nsLTP-syndrome due to cross-reactivity with homologous allergens contained in pollen, seeds, and fruits. Ara h 10, Ara h 11, Ara h 14, and Ara h 15 belong to oleosins, a lipophilic family of proteins associated with a severe allergy to peanuts [22]. Ara h 12, and Ara h 13 are defensins. Defensins are relevant allergens in Asteraceae pollen but are also identified in peanuts, soy, and celery.

### 4.2. Soybean

The soybean (*Glycin max*) is a common ingredient in many processed and pre-packaged foods. It contains a high quantity of high-quality proteins, up to 400 g/kg of soy. The protein content accounts for 36–56% of the total soybean content, and the oil content is about 200 g/kg of soybeans. The soybean also contains several bioactive molecules, such as tocopherols, isoflavones, phytates, phytosterols, lunatic, and saponins [60]. Soybean protein has an amino acid composition similar to proteins of animal origin, particularly whey protein. For this reason, soybeans have acquired considerable importance as a source of dietary protein, but significant drawbacks regarding the allergenicity and digestibility of their proteins still need to be addressed. Soy can be found as an ingredient in tofu, fermented soybeans (natto), babies’ formula milk and other dairy products, bakery goods and pastries, spreads, sauces, and as an additive in many processed foods, as well as in various pharmaceutical and industrial preparations. Therefore, its avoidance is challenging for patients with soy allergies [61]. Only 50% of children affected by soy allergy outgrew their allergy by the age of seven years [20,62]. Soy allergy has been reported to appear in adulthood, too, especially in people with persistent peanut allergy or birch-pollen allergy [63]. Sixteen different proteins have been identified in soybeans [64]. Still, only eight proteins have been registered with the World Health Organization and International Union of Immunological Societies (WHO/IUIS) Allergen Nomenclature Subcommittee [65]. The primary sensitizers involved in immediate-type allergic reactions are the seed storage proteins Gly m 5 or Beta-conglycinin (vicilin, 7 S globulin) and Gly m 6 or Glycinin (legumin, 11 S globulin) belonging to the cupine superfamily, and Gly m 8 (2S albumin) from prolamine superfamily. This allergen presents homology with Ara h two from the peanut. These allergens are associated with severe food allergies in children and adults [21,66]. Gly m 4 (PR10) cross-reacts to Bet v 1 and is implicated in pollen food allergy syndrome (PFAS). Moreover, Gly m 4 represents a risk factor for severe reactions to soybeans in subjects sensitized to the major allergen of birch pollen [67]. Gly m 1 (hydrophobic protein from soybean) and Gly m 2 (defensin) are, respectively, abundant in soy dust and husk and have been associated with asthma after inhalation [67]. Gly m 3 is profilin and shows cross-reactivity with Bet v 2, a profilin from birch pollen. Some lectins from legumes are not included in the IUIS-approved list of allergens but have a potential allergenic role to be investigated. Lectins or carbohydrate-binding proteins, similar to genuine legume allergens, can bind IgE from allergic subjects, induce the degranulation of sensitized basophils, and promote interleukin secretion in sensitized people. Soy lectin is SBA (soybean agglutinin) and accounts for 2% of the total protein content. Other legume lectins that are non-IUIS approved are Ara h agglutinin (*Arachis hypogaea*), LcA (*Lens culinaris*), PHA-E e PHA-L (*Phaseolus vulgaris*), PsA (*Pisum sativum*), and Chia lectin (*Salvia hispanica*) [68]. Moreover, in murine models, it has been demonstrated that isoflavones (genistein, daidzein, and glycitein), bioactive compounds referred to as phytoestrogens, can suppress the allergic response to peanuts. Anti-inflammatory isoflavones are in large amounts in soybeans and not in peanuts, which may explain why soy is less allergenic than peanuts. This evidence supports further studies to identify the food characteristics that promote the immune response and strategies to prevent food allergies [69].

### 4.3. Lupine

Lupine (Lupineus) includes four edible and nutritionally relevant species, also known as “sweet lupines” because they are low in alkaloids, namely the white lupine (*Lupineus albus*) native of the Mediterranean region and Africa, the blue lupine (*Lupineus angustifolius*) from Australia, yellow lupines (*Lupineus luteus*) from Central Europe, and pearl or Andean lupine (*Lupineus mutabilis*) from South America [69]. The food industry typically uses white lupine, while yellow and blue lupine are used for animal feed. Lupine has been eaten in the Mediterranean area for ages. Since the 1990s, lupine flour has been increasingly used as an ingredient or additive because of its nutritional value and technological properties, but it is also used in dermo-cosmetic products [70]. Moreover, high-protein diets are a trend in dietetics and health nutrition as an approach to weight loss. So, diets containing legumes are gaining popularity as a source of protein [71]. The use of lupine flour as a substitute protein source in bakeries, pasta, vegetarian or gluten-free products, and other food items (non-animal dairy products and considered for plant-based meat analogs and baby-food formulations) has grown in the last few years [72]. Lupine has a high content of protein (32–38%), dietary fiber, antioxidants, and vitamins and low levels of fatty acids and carbohydrates [73]. The nutritional value of lupine proteins is attributable to high concentrations of the essential amino acids lysine, leucine, and threonine, which are higher only in soybeans [74]. The anti-nutritional compounds include alkaloids (the content in sweet white lupine crops has been reduced in the cultivation process and is currently below 0.02%), inhibitors of proteases, saponins, phytic acid, and lectins, which are present in small amounts [73]. The safety features of lupine ingredients concern the formation of biogenic amines and the presence of allergens [75,76]. Lupine allergy can present as a primary allergy to lupine or secondary to cross-reactivity with other legumes, mostly peanuts, with reactions ranging from urticaria and vomiting to severe anaphylaxis [9,25,76]. Lupine allergy is related to two sensitization pathways, namely primary sensitization occurring through ingestion and secondary to inhalation, consequent to occupational exposure or pollen-associated lupine allergy (Bet v 1 birch-pollen-related proteins) [72]. The major allergens of the Lupineus species are storage proteins, namely the conglutins. The fractions are α-conglutin (11 S globulin or legumin-like, accounting for 33%), β-conglutin (7S globulin or vicilin-like, accounting for 43.4%), γ-conglutin (7S globulin or vicilin-like, accounting for 6%) from the cupine superfamily and δ-conglutin (2S albumins, accounting for 12.5%) from the prolamine superfamily. There are some minor protein fractions, such as pathogenesis-related (PR)-10 proteins, nonspecific lipid-transfer proteins (nsLTP), and profilins (Lup a 5) [72]. The Lupine angustifolius allergen β-conglutin, characterized as a major allergen, denominated Lup a 1 by IUIS Allergen Nomenclature Subcommittee, cross-reacts with Ara h 1 from the peanut and Gly m 5 from soy, while Ara h 2 and Gly m 8 with δ-conglutins and Ara h 3 only showed inhibition of IgE binding to α-conglutins at the highest concentrations [77]. Recently, a study illustrated the role of γ-conglutin as a major lupine allergen in a population from Chile [69].

### 4.4. Pea

Peas (*Pisum sativum*) are legumes commercially presented as unripe pods (peas and snow peas), green unripe seeds (garden peas), and dried ripe peas (field peas or duns), the latter characterized by various colors, including brown, yellow, green, and purple [78,79]. Peas are a source of plant-based protein; dry garden peas and field peas contain about 25% of proteins. Garden peas are typically eaten fresh, with a 4–5% protein content. Cooked peas contain about 8% protein. The field peas’ protein content also depends on the maturation and fermentation of seeds, and it can be as high as 30% depending on the variety, climatic conditions, and other factors [79]. Green pea (*Pisum sativum*) and Dun pea (*Pisum sativum* var. *arvense*) are the most clinically relevant. Green peas are commonly eaten, even raw, and dun peas are frequently used in the manufacture of other foods as a source of proteins. Dun pea proteins are used as flour or an isolate as an alternative to lupine flour or soy protein, in plant-based dairy alternatives, processed meat, cookies, gluten-free and vegan products, protein-fortified meals for athletes, and pharmaceuticals. Pea proteins are not mandatorily labeled but are generally addressed as vegetable proteins, thus exposing consumers to the potential risk of food allergies and anaphylaxis from hidden allergens [79,80]. The pea allergens recognized by the International Union of Immunological Sciences (IUIS) are Pis s 1 (Vicilin), Pis s 2 (Covicilin), and Pis s 3 (ns-LTP). Pis s 1, and Pis s 2 are storage proteins proposed as major pea allergens [80]. Pis s 3 could be responsible for FDEIA, as previously reported by a study on adults and children [36]. There are two additional allergens, Pis s 5 (Profilin) and Pis s 6 (Bet v 1-like), yet to be included in the IUIS/WHO database [49]. Pis s 1 shares 50% homology with Ara h 1 from the peanut, with Lup a 1 (52%) from lupine, Gly m 5 (52%) from the soybean, and 90% with Len c 1 from the lentil without an established clinical relevance in the latter case [21,81]. Pea cross-reactivity with other legumes, particularly peanuts, was confirmed by different studies. About 64% of pea-allergic children presented a parallel other legume allergy; the most frequent allergen involved was peanut (77%). Moreover, a Canadian report described six children with anaphylaxis due to peas containing a hidden allergen, four of whom were also allergic to peanuts [37,81]. Some reports in the literature of children allergic to cooked peas but tolerating the raw ones suggest that heating could increase pea allergenicity [82]. Therefore, it is important not to exclude a legume allergy only based on tolerance to raw legumes [83].

### 4.5. Chickpea

The chickpea (*Cicer arietinum*) is the most consumed legume worldwide and is considered one of the oldest cultivated legumes in Asia and Europe. There are two types of cultivated chickpeas, namely Desi, grown in Asia and Africa, and Kabuli, grown in Europe, North Africa, North America, and West Asia [84,85,86,87]. Chickpea allergy has been described chiefly in children living in the Mediterranean area and in the Indian population [32,35,84,85,86,87]. Chickpeas, or garbanzo beans, are incorporated in hummus, falafel, soups, snack mix, crackers, and bread made with chickpea flour. The chickpea is an excellent protein, carbohydrate, fibre, essential minerals, and vitamin source. The chickpea protein content on a dry mass basis varies before (17–22%) and after dehulling (25–29%) and between the two types, Kabuli and Desi. In chickpeas, globulins are the dominant storage proteins and account for 50–63% of seed protein, followed by albumins (12.0%), glutelins (18%), and prolamins (3–7%) [84,85,86,87]. There have been identified four chickpea allergens, namely Cic a 1 (7S vicilin), the only one included in the IUIS/WHO allergen database; Cic a 2S albumin (2S albumin); Cic a 3 (LTP); and Cic a 6 (11S globulin) [49]. Cic a 1 showed cross-reactivity with SSP from lentils, peas, soybean, and hazelnuts. Interestingly, chickpea allergy is never isolated, being more commonly associated with lentil and pea allergy [84,85,86,87]. Clinical cross-reactivity between these three legumes was often observed in Spanish subjects [21]. Moreover, two cases of FDEIA in chickpeas have been reported in adolescents [87,88].

### 4.6. Lentil

The lentil (*Lens culinaris*) is a legume and an important food source in children’s diets. Lentils are frequently consumed in the Mediterranean, Middle East, Asia, and North America. They are Turkey’s sixth most common food allergen and the fourth in the Spanish pediatric population [32,89]. The commercially available lentils vary in size, coat color (green, brown, grey, purple, or black), and seed cotyledon colors (yellow, red, or green). Common lentil market classes include red, green, yellow, and Spanish brown. Red and green lentils are the most consumed. Red lentils are usually dehulled before cooking, while green lentils are consumed dehulled or as whole seeds [90]. Lentils are included in many regional gastronomies throughout the world (Indian curry, salads, and lentil or legume soup), fortified yogurt, and energy bars, but also in bakery and extruded products (such as snacks and pasta). It is valuable for a vegan or gluten-free diet [91]. Lentils are a source of protein (22–26% of dry weight), complex carbohydrates, vitamins, dietary fibre and minerals. According to the WHO/IUIS Allergen Nomenclature Sub-Committee, there are three lentil allergen proteins, namely Len c 1 (vicilin), Len c 2 (seed-specific biotinylated protein), and Len c 3 (ns-LTP). Approximately 80% of the patients with lentil allergy identify the purified Len c 1 protein [92]. A recent study on children from Turkey with legume allergy showed that lentil was the most frequent culprit allergen (66%), followed by peanuts (61%), chickpeas (28%), peas (24%), beans (8%), and soybean (1%). About 60% of patients had multiple (≥2) legume allergies, with symptom onset earlier (median 18 months) than in subjects with a single legume allergy (median 28 months). The highest incidence of anaphylaxis was observed with lentils (34%) and peanuts (32%). Moreover, allergic reactions after exposure to the cooking steam of lentils or other legumes have rarely been described [93]. It has already been mentioned that there is wide cross-reactivity among lentils, chickpeas, and peas in the Mediterranean area due to sensitization to allergenic molecules/epitopes, such as Len c 1 from lentils, Pis s 1 from peas, and the globulin fraction of chickpeas [8].

### 4.7. Bean

The common bean (*Phaseolus vulgaris*), also known as the French bean, haricot bean, salad bean, snap bean, and string bean, is a member of the Fabaceae family. The legume is available as edible dry seeds or green, unripe pods. Many varieties, such as navy beans, kidney beans, red beans, black beans, pinto beans, and cranberry or Roman beans, belong to this species [94]. Dry beans are usually processed before eating, generally by soaking and cooking in water, but some beans are consumed after roasting or milling into flour. Like other legumes, beans showed a potential for producing extruded foods and snacks and plant-based meat alternatives [94,95]. The protein content ranges between 15% and 35%, whose fractions are globulin (50–70%) and albumin (10%). Globulins are divided into 7S and 11S, and the 7S fraction named phaseolin (Pha v) is a vicilin and the major seed storage protein described in red and white kidney beans. The 11S globulin fraction of legumin is only 10%, but it has also been reported as a major bean allergen responsible for bean (pinto bean) allergy [96]. Prolamine and glutelin are less represented [94]. Furthermore, in green beans, another allergen related to a major avocado allergen has been recognized as Pha v Chitinase. In red kidney beans, a major allergen, a phytohemagglutinin (Pha l), was isolated with cross-reactivity to peanut and black gram. Moreover, profilin (Pha v 5), Bet v 1–like allergen (Pha v 6), and lipid-transfer protein (LTP-Pha v 3) have been identified in the common bean and presented a high rate of cross-reactivity with other legumes and vegetables. The Pha v 3 is the only allergen in the IUIS/WHO database [96]. Although the bean is usually well tolerated, even in children allergic to other legumes, there have been a few reports of bean allergy in France, Spain, and Japan [8,96,97,98,99]. A food allergy to a less common legume, e.g., beans, suggests a high allergic predisposition, and the presence of bean allergy could be a sign of multiple legume allergies [8].

### 4.8. Faba Bean

Faba bean (*Vicia faba*), or horse or broad bean, represents an excellent source of high-quality dietary proteins (about 30%). Faba bean proteins are represented by a globulin fraction (about 70%) divided into two major types, namely legumins, composing up to 55% of the protein, and vicilins. To a lesser extent, glutelins (12.0 to 18.4%), prolamins (1.83 to 3.57%), and albumins (1.41 to 3.01%) are present [100]. Faba bean proteins, similar to other legumes, can potentially induce allergic reactions. A recent study describing the prevalence of sensitization to various legumes in a group of allergic patients showed that faba bean sensitization prevalence was among the lowest (5.7%) [101]. There were some reports of allergic reactions to faba beans in the literature, also as a hidden allergen used in presliced bread as an additive [43,44,102]. Faba bean may also cause favism, another adverse health effect quite common in the Mediterranean area and mistaken for allergy by patients. Favism is hemolytic anemia due to a deficiency of glucose-6-phosphate dehydrogenase (G6PD) transmitted as a recessive X-linked trait. The G6PD deficiency, in the presence of oxidative imbalance caused by isoamyl and divicine from the faba bean, leads to oxidative stress on erythrocytes and consequent hemolysis [103].

### 4.9. Fenugreek

Fenugreek (*Trigonella foenum-graecum*) belongs to the Leguminosae family. It is a plant-based food in Iran, India, Ethiopia, Canada, Oman, and Turkey. The fresh leaves and the dried seeds, whole or as flour after roasting, are used for food purposes and in traditional medicine [104]. The medicinal properties of fenugreek, known since ancient times, include hypocholesterolemic, antidiabetic, antihypertensive, gastric stimulant, galactagogue, hepatoprotective, and anticancer effects. These beneficial actions, including the antidiabetic and hypocholesterolemic properties, are due to the micronutrients and high fibre content with nutraceutical value [105]. Fenugreek can be used in food products as a flavor/spice, food stabilizer, and emulsifying agent. It is found in cheese, baked goods, pasta and pizza, spices (especially curry), coffee alternatives, and teas, as well as in traditional recipes (e.g., cornbread with fenugreek in Egypt, Panch Phoron in Bengal, and Sambar in South India). It has currently gained attention for producing healthy and functional foods and for pharmaceutical purposes. Fenugreek contains 23–26% of proteins and 58% of carbohydrates, 25% of which is dietary fibre [39,49,106]. Cases of fenugreek allergy have been reported, including anaphylaxis and occupational asthma, after ingestion, inhalation, and external application of fenugreek-seed powder or curry intake both in adults and pediatric age [107,108,109,110]. Data from the literature describe that allergic reactions to fenugreek are often related to a primary peanut allergy, thus suggesting the existence of a cross-reactivity between peanut and fenugreek allergens. The Norwegian Register and Reporting System for Severe Allergic Reactions to Food reported that reactions to spicy foods and Indian meals in peanut-allergic subjects were due to fenugreek [111]. Nevertheless, some cases of primary allergy to fenugreek occurring in subjects with another legume allergy (lentil, faba bean, and chickpea allergy) have been rarely reported [107,110,112]. Fenugreek allergens have been identified, namely Tri f 1 (7S vicilin), Tri f 2 (2S album].in), Tri f 3 (11S legumin), and Tri f 4 (PR-10 protein), but have not yet been included in the WHO/IUIS database [49]. The cross-reactivity between fenugreek and peanut might be due to the structural similarity between the fenugreek and peanut allergens. In particular, a proteomics analysis showed that Tri f 1 had homology to Ara h 1 (peanut), Pis s 1 (pea), Len c 1 (lentil), and beta-conglutin (lupine) and Tri f 2 with Ara h 3 (peanut) [113].

### 4.10. Tamaridus

Tamaridus (*Tamaridusus indica*) is a legume belonging to the sub-family of Caesalpinioideae and is broadly distributed in tropical countries, such as sub-Saharan Africa, India, Pakistan, and Bangladesh. The Tamaridusus has been used for both herbal, medicinal, and food purposes for a long time. In traditional medicine, Tamaridusus exerts anti-inflammatory and analgesic effects due to bioactive compounds, like flavonoids, alkaloids, tannins, phenols, triterpenoids, fatty acids, saponins, and steroids [112]. The fruit is a brown pod containing a bittersweet pulp with seeds inside. The pulp is used as a food component in curries and sauces (e.g., Worcester sauce and Indian chutney), juices, and certain refreshing drinks (e.g., those typical in Africa and Sicily) [111]. From the seeds derives the tamaridus kernel powder (TKP) used in the food industry (as a gelling, thickening, and preservative agent), textiles, pharmaceuticals (drug delivery system), cosmetics, and other industrial applications (as an adhesive agent). Jellose or polyose is a polysaccharide isolated from TKP with similar properties to fruit pectin. So, TKP as a gelling, preservative, or additive agent could be found in desserts, jellies, chewing gum, ketchup, jams, pancakes, confectionery, yogurt, noodles, soybean curds, breads, gluten-free products, and so on [114,115,116,117,118,119]. Despite its widespread use in food, tamaridus and its byproducts are not subject to mandatory labeling [12]. Tamaridus seeds are good sources of protein, fibre and carbohydrates. The protein content of tamaridus seeds is around 23.6%. The crude extract of TKP contains two major proteins, identified as a class III chitinase (the major storage protein named “tamarinin” accounts for >50% of soluble proteins) and a trypsin inhibitor [114,115,116,117,118,119]. In general, allergenic chitinase can be found in mites; cockroaches; and fruits, such as bananas, papaya, pomegranate, avocado, and latex [21]. Many plant chitinases have a structural similarity with hevein from latex, with a possible cross-reactivity syndrome [118]. In general, the onset of sensitization through the oral route to the gums is unusual, but sensitization by inhalation has been reported [120]. Some previous studies described an occupational allergy to tamaridus flour and seeds [114,115,116,117,118,119].

### 4.11. Legume-Based Food Additives

Other food additives as thickening and stabilizing agents that are derived from legumes include gum Arabic (E414) from *Acacia* spp., guar gum (E412) from Cyamopsis tetragonoloba, locust bean/carob bean gum (E410) from Ceratonia siliqua, and Tragacanth gum (E413) from Astragalus gummifer. They are polysaccharides used as additives in foods such as sauces, baked foods, salad dressings, thickened milk products, yogurts, canned soups, etc. Reactions to these gums are most often linked to occupational allergy, even if there have been case reports of an anaphylactic reaction to guar gum after its ingestion [120,121,122,123]. Occasionally, carob gum was also associated with allergic reactions after the ingestion of food containing it as an additive. A study on possible clinical cross-reactivity between carob and peanuts reported that peanut-allergic children tolerated cooked carob seeds [124,125,126].

## 5. Effect of Legume Processing

Legume processing acts at three levels, namely preservation and detoxification by reducing the presence of anti-nutritional factors (ANFs) and possibly modifying the allergenicity of proteins. Legumes include numerous bioactive compounds, commonly called ANFs, because they decrease the digestibility of proteins and carbohydrates and the bioavailability of vitamins and minerals. These ANFs are proteases (trypsin, chymotrypsin, and α-amylase inhibitors), saponins, lectins, total phenolics (tannins and flavonoids), and phytic and oxalic acid. Some ANFs have recently been shown to produce health benefits at low concentrations, such as antioxidant and anti-inflammatory activity [1,91]. In general, food processing may induce the modification of the conformational structure of the protein, thus inhibiting the binding of immunoglobulin E (IgE) to epitopes on food allergens and, consequently, allergic reactions. Food-processing technologies employed for inactivating allergenic epitopes used thermal and non-thermal procedures [59,127]. Legumes are usually consumed after thermal processing, which reduces/removes ANFs and may influence protein allergenicity. The treatments most commonly applied in domestic settings are soaking, cooking (traditional, microwave, and pressure), and baking, whereas industrial processing comprises autoclaving, baking, and extrusion [128]. A study was conducted on a cohort of 106 patients > 16 years with a suspected legume allergy and positive for SPT, PBP, and sIgE. It evaluated sensitization for legume extracts before and after processing (legumes were soaked for up to 24 h and cooked (100 °C) in water for 20–100 min, and peanuts were roasted in a hot air oven for 12 min at 175 °C). The prevalence of sensitization for the non-processed legumes showed the highest prevalence of sensitization for peanut (14.2%), white lupine (13.2%), green pea (9.4%), blue lupine (8.5%), soybean (8.5%), chickpea (8.5%), and white bean (7.5%), with a lower prevalence for black lentil (6.6%), faba bean (5.7%), and green lentil (5.7%). After processing, an increased prevalence of sensitization was observed for peanut (16.0%) and soybean (10.4%), with a decreased prevalence of sensitization for white lupine (8.5%) and green pea (4.7%) [101]. The processing of peanuts with dry methods, such as microwaving, roasting, and frying at high temperatures likely promotes the formation of neoepitopes. It increases the allergenicity of Ara h 1 and Ara h 2 and the oleosins, whereas cooking with wet methods, boiling, and high-pressure steaming/autoclaving might decrease their allergenicity [21,129]. Soybean allergenicity can be modified by thermal processing, enzymatic degradation, and fermentation [127]. Soybeans boiled for 15 and 30 min are not associated with a significant reduction in the IgE-binding protein profile, while autoclaving gradually reduces IgE-reactive proteins [130]. In the industrial setting, high hydrostatic pressure (HHP) has been reported to be an effective method to attenuate the allergenicity of legumes, particularly soybeans and peanuts [131]. Lupine allergenicity is reduced by autoclaving (138 °C for 20 min), while boiling, roasting, microwaving, and extrusion cooking do not influence allergenicity [70]. The processing of lentils through boiling induced little to no modifications in the IgE-binding profile, although autoclaving at 2.56 atm for 30 min significantly reduced the IgE binding of lentil allergens [7]. Extruded products from lentils, such as pasta, have an allergenic content similar to those in lentils. Lentil pasta and lentil seeds are equally impacted by boiling [132]. Similarly, the allergenicity of chickpea, pea, and bean protein is not influenced by boiling but is reduced through autoclaving. Interestingly, allergy to cooked peas and not to raw ones in six children has been described, signifying that cooking could increase pea allergenicity [83].

## 6. Clinical Manifestations of IgE-Mediated Legume Allergy

IgE-mediated reactions occur after ingesting legumes and rarely after contact with or inhaling cooking steam; some cases of occupational allergy have been described. Typical and reproducible symptoms in a sensitized subject generally develop within 2 h of exposure to the allergen, involving the cutaneous, neurological, cardiovascular, respiratory systems and gastrointestinal tract [133]. The symptoms range from mild to moderate (oral allergy syndrome, urticaria, rhinitis, angioedema, asthma, vomiting, and abdominal pain) to severe reactions, including anaphylaxis. In France, legumes are responsible for 14.6% of food-related anaphylaxis in children; peanuts represented the main allergen involved (77.5%) [134]. The incidence of anaphylaxis due to legumes has increased in the last few years, reaching 24.3% in the general population. Peanuts are the allergens that are the most frequent culprits, followed by soybeans, lupines, peas, lentils, beans, and chickpeas [24]. Rare allergic reactions to legumes after exposure to airborne food allergen particles have been reported. In particular, urticaria, angioedema, rhinoconjunctivitis, and asthma exacerbations have been described with the inhalation of the steam of lentils, soy, peanuts, chickpeas, and beans [93,135,136,137]. This evidence represents the expression of a secondary reaction following a first sensitization occurring through the oral route [93]. Airborne exposure is also associated with occupational asthma and rhinoconjunctivitis in workers manipulating lupine flour or during the preparation and cooking of raw green beans [138,139].

## 7. Diagnostic Approach

The diagnostic approach to legume allergy begins with a detailed medical history. Starting questions to be analyzed are the current age of the patient and age of onset of symptoms; additional allergic features (i.e., birch pollen allergy) or atopic diseases (i.e., atopic eczema and asthma); legume(s) involved in the reaction and latency time between ingestion or contact and onset of symptoms; contact and onset of symptoms (the number of episodes in which the patient has had symptoms should be recorded); treatment used in the reaction and the need for adrenaline; and the need for the emergency care. The contact method should be detailed, whether directly by ingestion, indirectly through skin contact, or the inhalation of cooking vapors. Symptoms generally develop after legume ingestion. However, reactions by contact or steam inhalation from cooked legumes have also been reported [3,135,140,141,142]. Symptoms of the reaction must be investigated, such as cutaneous signs (urticaria and angioedema), food refusal, oral allergy syndrome, gastrointestinal symptoms (diarrhea, vomiting, and abdominal pain), respiratory symptoms (bronchospasm, cough, dyspnoea, and wheezing), and rhino-conjunctivitis. After correlating the clinical symptoms with a convincing history of IgE-mediated reaction with a legume, in vitro and in vivo diagnostic tests should be applied. Skin-prick tests can be performed with commercial extracts of legumes or by a prick-to-prick technique using raw legume flour, ground raw legume seeds, or boiled legumes [143]. Although lentils, chickpeas, peas, and beans are consumed after cooking, food extracts commonly used for skin testing in clinical practice are prepared from crude materials [144]. Some authors described that skin tests with 15 min boiled extracts (0.5–5 mg/mL) were more appropriate to crude extracts for identifying children clinically sensitive to lentils [32,143]. Still, lentil and chickpea extracts maintain strong allergenicity after boiling [145,146]. Lentil extract treatment strongly increases bands corresponding to the major allergen Len c 1 in sodium dodecyl sulfate–polyacrylamide gel electrophoresis experiments. These data suggested that boiled extracts provide better predictive values than raw or commercial extracts and that thermostable allergens are responsible for legume allergy. Serum-specific IgE determination is available for chickpeas, white beans, green beans, peas, lentils, soybeans, lupine, fenugreek, and guar. A Turkish pediatric study evaluated the IgE cutoff levels to predict clinical reactivity to lentils at pediatric age. They speculated that, in the case of lentil-specific IgE values > 23 kU/L, the risk of an anaphylactic reaction after ingestion or exposure to lentil steam was remarkably high [147]. Moreover, the predicted probability curve for lentil sIgE determined that 17 kU/L was 95% to show a clinical reaction. Component-resolved diagnosis (CRD), the detection of serum-specific IgE against molecular components, has an important role as a second-level diagnostic step, although few legume allergens are still available. The following allergens are commercially offered only for soybeans and peanuts: Gly m4 (PR-10), Gly m 5 (7/8S globulin), Gly m 6 (11S globulin), Gly m 8 (2S globulin) and Ara h 2 (2S albumin), Ara h 6 (2S albumin), Ara h 8 (PR-10) 1, Ara h 9 (LTP). Lately, the basophil activation test (BAT) (functional assay using live basophils from allergic patients) has been observed as an excellent tool to establish the capacity of IgE to mediate the activation of basophils upon allergen stimulation [148]. It is still not used in routine analysis, but its application as a more reliable diagnostic test has been spreading. BAT proved to have a strong response to triggering legumes and others, although the patient previously tolerated those. Oral food challenges remain the gold standard for the diagnosis of legume allergy and for determining tolerance [133]. However, food challenges may occasionally result in severe clinical symptoms and should be carried out in hospital settings with emergency care availability. Specifically, during legume challenges, cutaneous symptoms, such as urticaria/angioedema and respiratory symptoms, have been the most observed [149]. When there is a suspicion of legume allergy, it is necessary to try to avoid a complete restriction of the group and to evaluate and assess the dietary habits of each family (Table 1).

Moreover, allergists often recommend restricting a patient’s diet to all members of a food family when the patient is affected by this type of food allergy [150]. It should be considered as carrying it out to introduce legumes, which are generally well tolerated by most patients (such as beans), considering alternatives rather than lifelong restrictions. Specifically, other legumes should also be tested in case of a positive diagnostic test to verify possible cross-reactivity phenomena. To confirm or reject the clinical significance of legume sensitization, an oral food challenge involving the specific type with less probability of association with allergic reaction should be performed [151].

## 8. Clinical Management

According to diagnostic test results, when the suspicion of IgE-mediated legume allergy is confirmed, the patient must avoid the ingestion of the triggering legume or products susceptible to containing it and other legumes [150]. In particular, contact and exposure to the sewing steamer should be avoided. It is necessary to remind the patient of the possibility of hidden allergens (especially peanut, soy, and lupine) in manufactured products [39]. Moreover, it should be kept in mind how to thicken and stabilize packaged foods (Arabic rubber E-414, tragacanth E-413, guar E-412, Algarrobo, or garron E-410), additives, and aids in the bread industry as a complement to bread (leguminous seeds such as broad bean, soy, lentils, and vegetables), emulgent (soy lecithin), and stabilizers (algarroba and guar flour). Though rare, reports of reactions to guar gum, tragacanth, licorice, and tamaridus must be considered. (Table 2)

**Figure 2:**
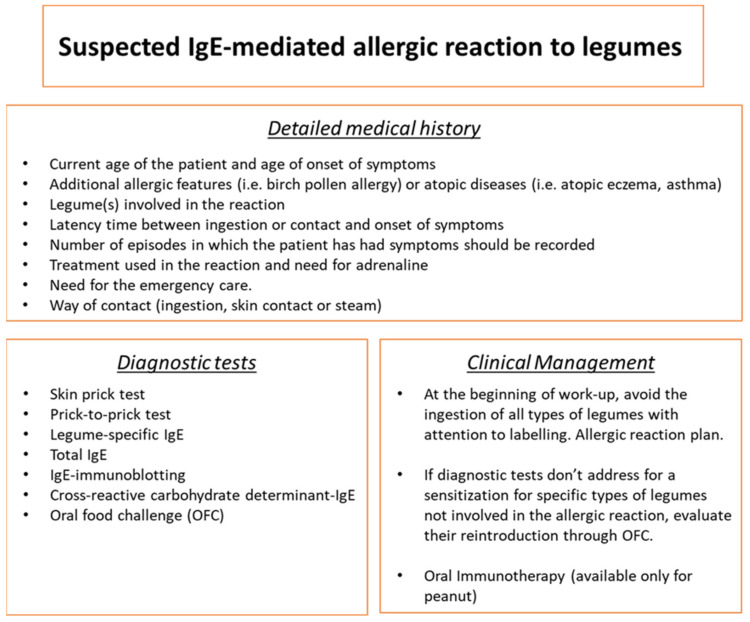
Proposal of diagnostic and clinical workup in legume IgE-mediated allergy in children.

The treatment tailored for accidental allergic reactions to specific clinical patterns depends on the risk of severe reactions after unintentionally ingesting legume-containing products [152]. In any case, self-administered emergency medication is required. In particular, due to the risk of severe reactions after the unintentional ingestion of legume-containing products, adrenaline for self-administration should always be on hand. Patients and families should be trained to treat auto-injectable adrenaline in severe clinical manifestations, explain the indications for use, recognize the symptoms of a reaction, and show how to use it [153]. Annual check-ups are recommended to repeat the diagnostic workup to assess the possibility of tolerance or sensitization to other foods [151]. Commercial products for allergen-specific immunotherapy to peanut allergy are commercially available [154]. The European Academy of Allergy and Clinical Immunology Guidelines on allergen immunotherapy specified that oral immunotherapy is recommended as a treatment option to enhance the threshold of reaction among children affected by peanut allergy from four to five years of age [155]. However, it should only be undertaken in highly specialized clinical centers with expertise and facilities [155] (Figure 2).

Currently, commercially available products for the allergen-specific immunotherapy of other legumes are unavailable [155].

## 9. Prognosis and Natural History

Legume allergy occurs during the first two years of life [8,32]. Most children manifest their allergic reactions on their first known exposure to a certain legume. Almost 60% of patients are allergic to more than one legume, since it may be related to the sensitization to cross-reactive allergenic molecules [147]. Moreover, most cases are part of a multiple food allergy. Some authors suggested that legume allergy reflects a strong atopic background [8]. Little is known about the prognosis of legume allergy in childhood. A Turkish study analyzed a possible decision point of specific IgE to lentils to predict the persistence of allergic symptoms. Children with an initial lentil sIgE < 4.9 kU/L had a significantly higher likelihood (68.4% vs. 18.2%) of outgrowing the lentil allergy than children with an initial lentil sIgE > 4.9 kU/L (*p* = 0.008) [147]. The same study determined that 50% of children developed tolerance before their teenage years [147]. Regarding peanuts, the persistence of this allergy showed a longer clinical reactivity, with a resolution that may occur during adolescence [155].

## 10. Conclusions

In recent years, the concern about legume allergy has fundamentally increased since more cases of relevant clinical manifestations have been reported after ingestion [7]. Despite the vast consumption of legumes and the growing frequency of legume allergy, few data concerning the characteristics of children with legume allergy are available [32]. Legumes, such as high biological value proteins and micronutrients, are often used for high nutritional properties [156]. Furthermore, legumes can be applied as a substitute for wheat in gluten-free products and as a technological aid in meat or dairy products for vegan diets [49]. From an allergological point of view, legumes contain potent allergens, such as seed storage proteins, that represent the major allergens responsible for allergic reactions in most patients. Diagnostic workup starts with a detailed clinical history and is supported by skin-prick tests, the prick-to-prick technique, and serum-specific IgE detection (Figure 2). Oral food challenge remains the diagnostic gold standard for legume allergy in children. Clinical management is still represented by the food elimination diet and an adequate action plan for allergic reactions, eventually involving the use of auto-injectable epinephrine. Anyway, the role of the pediatric allergist is to avoid only the specific type of legume involved in the allergic reaction, trying to demonstrate tolerance for other types of legumes. Although allergen immunotherapy for peanuts is commercially available with high therapeutic effects and improved quality of life, this therapeutic tool is not available for other legumes. Further research is needed.

## Figures and Tables

**Table 1:** Common foods containing legumes according to type of legume and category.

Category	Type of legume	Foods
Peanuts	Also called *Arachis hypogaea*, Beer nuts, Cacahuete, Chinese nuts, Earthnuts, Groundnuts, Goober nut/pea, Madelonas, Monkey nuts	Mueasli and cereals, cereal bars Unrefined oils, butters or spreads, Cosmetic products Praline and chocolate confectionery, cakes, biscuits, sweets, dips and ice creams, vegetarian food and desserts, satay sauce
Soybean	Edamame	Vegan/vegetarian and gluten-free foods (e.g. sausages, burgers) Soya milk or yogurt and desserts, ice creams, protein shakes and chocolate pudding Cheese substitutes and vegetarian meals (Tofu, miso, tempeh, Natto) Soya margarine and oil Breads and cereals, noodles pots Fruit products (eg dried fruit)
Lupine	Seeds, flour	Pastries, cakes, biscuits, pizza bases, gluten free products
Pea	Green, snow, sugar snap, mangetout, black eyed, pigeon pea, wasabi peas,	Processed meat (sausage, burger, hotdog, scotch eggs) Milk alternatives (e.g pea milk or alongside other milks such as oat milk) Bread, bagels, Indian style bread, Gluten free bakery products Vegan/vegetarian dishes, Sorbets, Soups, Bombay mix, Paella, risotto
Chickpea	Garbanzo bean or gram	Gram flour, Bhaajis, Pakoras, Hummus, Falafel
Lentil	Brown, green, red, puy, beluga, pardina	Poppodums (lentil flour), Dhal, Soups, Vegan/vegetarian dishes (lentil cottage pie), Lentil crisps
Bean	Adzuki, black, borlotti, broad (lima), cannellini, carob, flageolet bean, faba, French, green, haricot, kidney, lima, marrow, mung, pinto, runner, string, turtle	Tinned beans, Baked beans, Salads Chilli, Burritos Coconut milk (faba bean) Mediterranean dishes Vegan/vegetarian meals
Fenugreek, methi	Leaves, seeds	Curry powder, spice blends, Indian dishes

**Table 2:** Unexpected food involved in allergic reactions to legumes.

Guar gum	thickener and emulsifier (yoghurts and salad dressings)
Tragacanth	thickening agent and stabilizer in food products (salads and sauces) obtained from dried sap of several species in Middle Eastern legumes
Liquorice root	flavoring in confectionary, drinks and foods
Tamarind	produced from a brown bean pod and used as a paste, concentrate or powder to flavor food (chutneys and curries)
Carob bean	used as a thickening agent in anti-reflux formula

## Data Availability

Not Applicable.
